# Obituary

**Published:** 1889-10

**Authors:** 


### OBITUARY.

Elisha F. Thayer died on July 29th, 1889. Dr. Thayer was born at Dedham, Mass., in 1815. Although the son of a physician, and from early age was devoted to animals, he only commenced the study of veterinary medicine at a comparatively late period. In 1853 he visited Glasgow and London, and was an apt pupil of Professor Gamgee. In 1862 he was appointed a Cattle Commissioner of Massachusetts, and held that office for a quarter of a century. He was also for some years a member of the United States Cattle Commission. Dr. Thayer was one of the original members of the United States Veterinary Medical Association. After aiding in its organization in 1863, he served as censor for a number of years, as Treasurer, and as President in 1869, to which office he was re-elected in 1870. In 1869 Dr. Thayer received the degree of M. D. from the University of Vermont. He was a talented investigator, especially devoting himself to Contagious Pleuro-pneumonia, an authority on veterinary subjects and a member of the profession who did so much for it, that his loss will be mourned by his cotemporaries and remembered by their successors.

H.

---

CHARLES L. MOULTON, M.D.V.S., was born in Manchester, N. H., on the 29th day of May, 1849.

He was educated in the public schools in that city. At the age of eighteen he sailed for England, remaining about a year ; on his return he followed wholesale butchering business.

He was married in his 23d year, was then in retail meat business until 1879, at which time he contracted serious heart trouble by lifting quarters of beef ; he was in a short time obliged to give up business for a period of nine months.

He then proceeded to the American Veterinary College, New York City, to study the veterinary profession ; he graduated in 1882. He followed his profession in Manchester, N. H., until December 31st, 1883, when he received an appointment from the Secretary of War as Veterinary Surgeon to the Ninth Cavalry ; he was immediately ordered to Fort Reno, Indian Territory. He resigned his position on the last of June of the same year, or rather 1884. Then he took Dr. Holcomb's practice at Leavenworth, Kansas, remained there until he received another appointment from Lieut. Col. R. N. Batchelder, then at the head of the Depot Quarter Master's Office, at Washington, D. C., to take charge of the public animals owned by them. He also had charge of those at Ft. Myer and Washington Barracks; both public and private horses at the White House, and for a short time did the District work, which, together with his private practice, was very detrimental to his health, so he resigned the District work.

He graduated from the Georgetown University on the 8th of May, 1877, as an M.D. On the 15th of August he was ordered to St. Louis to buy and inspect horses for the Government; he remained there for nine months, then resigned his position, and returned to Washington, where he followed private practice, prior to his last sickness. He was taken sick on the 2d of March, and died on the 29th. A widow survives him. B.

